# Intellectual disorder type 98 caused by a novel NEXMIF variant: a case report and literature review

**DOI:** 10.3389/fmed.2026.1757682

**Published:** 2026-02-06

**Authors:** Yuanhang Zhu, Fangying Cui, Xuezhe Ouyang, Jing Guo, Yaming Liu, Naiqi Li, Qian Yang, Yali Li, Ling Liu

**Affiliations:** Department of Medical Genetics and Prenatal Diagnostics, The Third Affiliated Hospital of Zhengzhou University, Zhengzhou, China

**Keywords:** genetic counseling, intellectual disability type 98, NEXMIF, sanger sequencing, whole exome sequencing

## Abstract

**Background:**

Intellectual disorder, Type 98 (ID 98) is an X-linked disorder characterized by intellectual disability, epilepsy, and multisystem manifestations. This condition is caused by pathogenic variants in the NEXMIF gene through X-linked dominant inheritance.

**Case presentation:**

We identified a novel hemizygous NEXMIF variant (c.1939_1942delinsAT, p.S647Ifs*3) in a 5-year-old male with severe intellectual disability via trio whole-exome sequencing. His mildly affected mother was a heterozygous carrier. Prenatal diagnosis for the mother’s subsequent pregnancy identified the same hemizygous variant in the male fetus. Following genetic counseling, the decision was made to terminate the pregnancy, thereby preventing the clinical manifestation of the disease in the offspring.

**Conclusion:**

This report expands the NEXMIF mutational spectrum and underscores the critical role of genetic testing in achieving early diagnosis and informed reproductive counseling for families affected by this disorder.

## 1 Introduction

X-linked intellectual disability (XLID), which results from mutations on the X chromosome and accounts for 5–10% of intellectual disability cases in males, can be categorized as non-syndromic (isolated) or syndromic (with comorbidities) (1). While Fragile X syndrome (FXS) is its most prevalent form, about 200 other XLID syndromes have been characterized, involving pathogenic variants in over 150 genes (1, 2). This higher prevalence correlates with the enrichment of brain-expressed genes on the human X chromosome (3). Intellectual disorder type 98 (ID98, OMIM #300524) is a rare XLID caused by pathogenic variants in the NEXMIF gene (previously known as KIAA2022), which encodes a neuron-specific protein critical for synaptic formation and cognitive development (4). Since its initial identification in 2004 (5), NEXMIF-related disorders have been recognized as a significant cause of XLID, characterized by neurodevelopmental impairments including Intellectual disability, autism spectrum features, epilepsy, behavioral abnormalities, and multi-system involvement such as digestive and endocrine manifestations (6). Statistical analyses revealed NEXMIF variants in only one out of a cohort of 59 families with intellectual disability that were preselected for having *de novo* variants (7), and in 1 of 138 individuals from a Polish epilepsy cohort (8). Collectively, based on the reported data from these cohorts, pathogenic variants in the NEXMIF gene appear to be a relatively infrequent cause of the genetic etiology underlying both intellectual disability and epilepsy.

Here, we report a novel NEXMIF variant (c.1939_1942delinsAT, p.S647Ifs*3), identified through whole-exome sequencing and validated by Sanger sequencing, in a 5-year-old male with a presentation of severe intellectual disability and pronounced language deficits. This variant has not been previously reported in the databases (HGMD, ClinVar, DECIPHER, and PubMed), thereby expanding the mutational spectrum of ID98. Notably, this case originated from a family seeking reproductive counseling. Amniocentesis during a subsequent pregnancy identified the same variant in the male fetus. Following genetic counseling, the decision was made to terminate the pregnancy, thereby preventing the clinical manifestation of the disease in the offspring. This scenario exemplifies the pivotal role of molecular diagnostics in prenatal risk management and birth defect prevention. Furthermore, we conducted a comprehensive literature review on variants of this gene, encompassing previously reported variant types, prenatal and postnatal clinical manifestations in patients, laboratory findings, and pathogenic mechanisms. This provides clinicians and researchers with a valuable reference for understanding the disorder, facilitating early diagnosis, and enabling birth defect prevention.

## 2 Materials and methods

### 2.1 Patients and clinical investigations

A pregnant woman with mild intellectual disability, presenting with normal language and motor function, who had previously given birth to a boy with severe intellectual disability and pronounced language deficits in 2018, presented at 24 weeks of gestation to the Department of Medical Genetics and Prenatal Diagnosis at the Third Affiliated Hospital of Zhengzhou University for amniocentesis-based prenatal diagnosis. No other family members were reported to have similar intellectual or developmental concerns. A signed informed consent was obtained from the parents. The studies were approved by the Ethics Committee of The Third Affiliated Hospital of Zhengzhou University (2025-368-01).

### 2.2 Whole-exome sequencing

Whole-exome sequencing (WES) was performed using the Illumina NovaSeq 6,000 platform. Raw sequencing data were processed with CASAVA v1.82 software. The quality of the sequencing data was rigorously assessed, meeting the following criteria: a mean coverage depth of >100×, with over 95% of the target bases covered at ≥20× and over 90% at ≥30×. The mapping rate exceeded 98%, and the on-target rate was greater than 65%. Additionally, the Q30 base ratio was >85%, and the duplication rate was <30%. High-quality sequencing reads were aligned to the human reference genome (hg19/GRCh37) using Burrows-Wheeler Aligner (BWA). PCR duplicates were removed with Picard v1.57 (http://picard.sourceforge.net). Variant calling was performed using Berry Genomics’ Verita Trekker® variant detection system and GATK v4.2.6.1 (https://www.broadinstitute.org/gatk/). Variant annotation was conducted via ANNOVAR (9) and Berry Genomics’ Enliven® annotation system.

To identify the causative variant, a stepwise filtering strategy was applied. First, variants were filtered based on population frequency using the gnomAD database (v2.1.1), retaining only those with an allele frequency of <0.1% for autosomal and <1% for X-linked variants. Given the patient’s phenotype and the noted family history, we prioritized variants under autosomal dominant, autosomal recessive, and X-linked inheritance models. Co-segregation analysis within the family was performed where DNA samples from relevant family members were available. Subsequently, we focused on non-synonymous variants, splice-site variants, and indels predicted to be deleterious by multiple in silico prediction tools. The filtered variant list was then cross-referenced with clinical and disease databases, including ClinVar, DECIPHER, the Human Gene Mutation Database (HGMD) and Pubmed to assess their previously documented pathogenicity and phenotypic associations.

### 2.3 Sanger sequencing

The WES-identified variant was validated by Sanger sequencing. Primers targeting the NEXMIF gene exon3: c.1939_1942delinsAT locus were designed as follows: Forward primer: CAGAGAAACACCAACACGGACT；Reverse primer: GCACAGCTAGGAGCACCCA; Amplicon length: 278 bp; PCR amplification and product purification were performed using standard protocols. Sequencing reactions were conducted with the ABI PRISM® BigDye® Terminator v3.1 Cycle Sequencing Kit, followed by post-reaction purification. Capillary electrophoresis was carried out on an ABI 3730 Genetic Analyzer, and results were analyzed using Variant Reporter v1 software (Thermo Fisher Scientific).

### 2.4 Fluorescent PCR combined with capillary electrophoresis

To rule out Fragile X syndrome, a common cause of male intellectual disability, the proband was tested by targeting the CGG triplet repeat expansion in the FMR1 gene. DNA was first extracted from the samples, followed by PCR amplification. The amplified products were then separated using the ABI 3500Dx Genetic Analyzer through a 50 cm capillary in long fragment microsatellite analysis mode (Fragment) with POP-7 polymer under an injection time of 30–40 s. Data analysis was performed using GeneMapper Software.

## 3 Results

### 3.1 Case report

**Figure 1:**
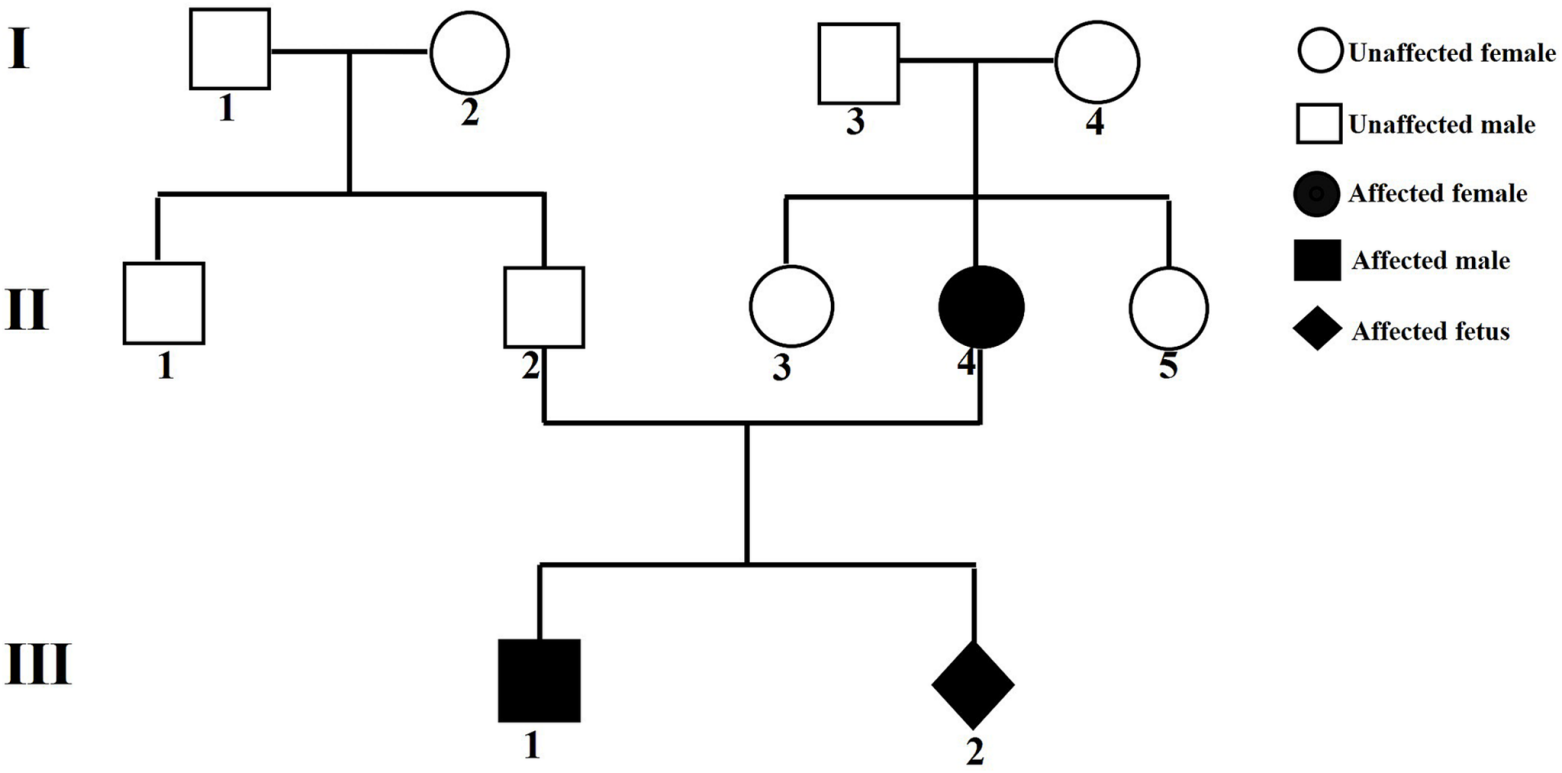
Pedigree of the family with the NEXMIF c.1939_1942delinsAT variant. II2: pregnant woman’s spouse; II4: pregnant woman; III1: proband; III2: current pregnancy fetus.

A mildly intellectually disabled gravida delivered a male infant (proband) at term via spontaneous vaginal delivery in 2018. The pregnancy course was uncomplicated, with a birth weight of 3.75 kg. Immediate neonatal adaptation was evidenced by vigorous crying. At 5 months and 7 days of age, the infant manifested persistent bilateral hand clenching and delayed psychomotor responsiveness, prompting hospitalization at the Third Affiliated Hospital of Zhengzhou University. During the initial evaluation, cranial magnetic resonance imaging (MRI) was reported to show no significant abnormalities. Nevertheless, the patient received a provisional diagnosis of “neurodevelopmental delay” and underwent multiple courses of neurorehabilitation, which yielded partial clinical improvement. By 8 months of age, the child exhibited global developmental delay characterized by failure to achieve gross motor milestones (inability to lift the head or roll over), absence of purposeful grasping, and markedly blunted environmental responsiveness. The child was readmitted for further rehabilitation with minimal functional gains, after which the family discontinued hospital-based rehabilitation. At 5 years old, the proband exhibited severe intellectual disability (as formally assessed with the Wechsler Intelligence Scale for Children), profound language impairment (largely nonverbal with only sporadic vocalizations such as “mama/papa”) and growth retardation (height below the third percentile). He was unable to follow simple verbal commands (e.g., “turn on/off the lights”) but retained the ability to walk independently, although he could not run or jump. Notably, the proband has never exhibited epileptic seizures and, consequently, electroencephalography has not been performed. In 2023, the proband’s mother became pregnant again and presented to the Department of Medical Genetics and Prenatal Diagnosis at 24 weeks and 6 days of gestation for prenatal consultation due to her adverse perinatal history. Medical geneticists performed family trio WES and Sanger sequencing on the pregnant woman, her spouse, and the proband. Based on the findings, prenatal validation of the pathogenic variant was conducted for the current pregnancy. The pedigree is presented in Figure 1.

### 3.2 Genetic analysis

**Figure 2:**
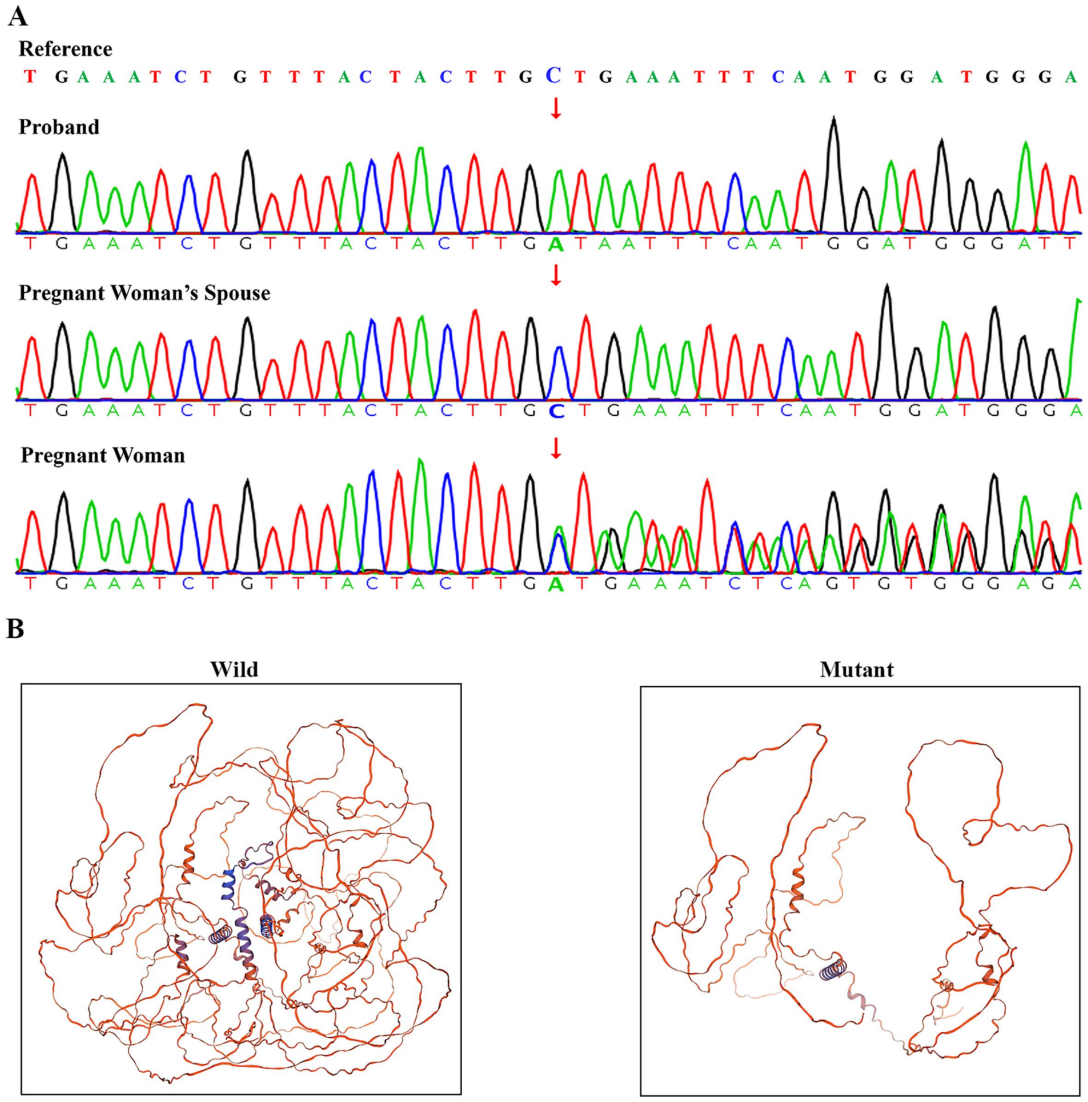
Verification and pathogenic structural analysis of the NEXMIF c.1939___1942delinsAT variant. **(A)** Sanger sequencing confirmation of the NEXMIF c.1939___1942delinsAT variant. **(B)** Comparative homology models of wild-type and mutant NEXMIF protein structures (c.1939___1942delinsAT) predicted using Swiss-model.

Family-based WES and Sanger sequencing identified the NEXMIF c.1939_1942delinsAT (p.S647Ifs*3) variant in a hemizygous state in the male proband and in a heterozygous state in his mother, consistent with X-linked inheritance. The woman’s spouse showed no NEXMIF variants (Figure 2A). Prenatal testing confirmed the fetus carried the hemizygous NEXMIF c.1939_1942delinsAT (p.S647Ifs*3) variant (Supplementary Figure S1). Per the American College of Medical Genetics and Genomics guidelines and ClinGen Sequence Variant Interpretation recommendations, the NEXMIF c.1939_1942delinsAT variant was classified as likely pathogenic based on: PVS1 (Very Strong): The variant introduces a premature stop codon, predicted to result in loss of function, a known mechanism of NEXMIF pathogenicity (Figure 2B). PM2_Supporting: The variant was not found in the human exome aggregation consortium (ExAC) browser, the 1,000 Project Genomes (1000G) Phase 3 and the genome aggregation database (gnomAD) v2.1.1. A minor allele frequency (MAF) filter of <0.0001 was applied to exclude common polymorphisms. At the time of preparing this report, comprehensive searches of the HGMD, ClinVar, DECIPHER, and PubMed databases, have not documented any reports of the NEXMIF gene variant c.1939_1942delinsAT.

**Figure 3:**
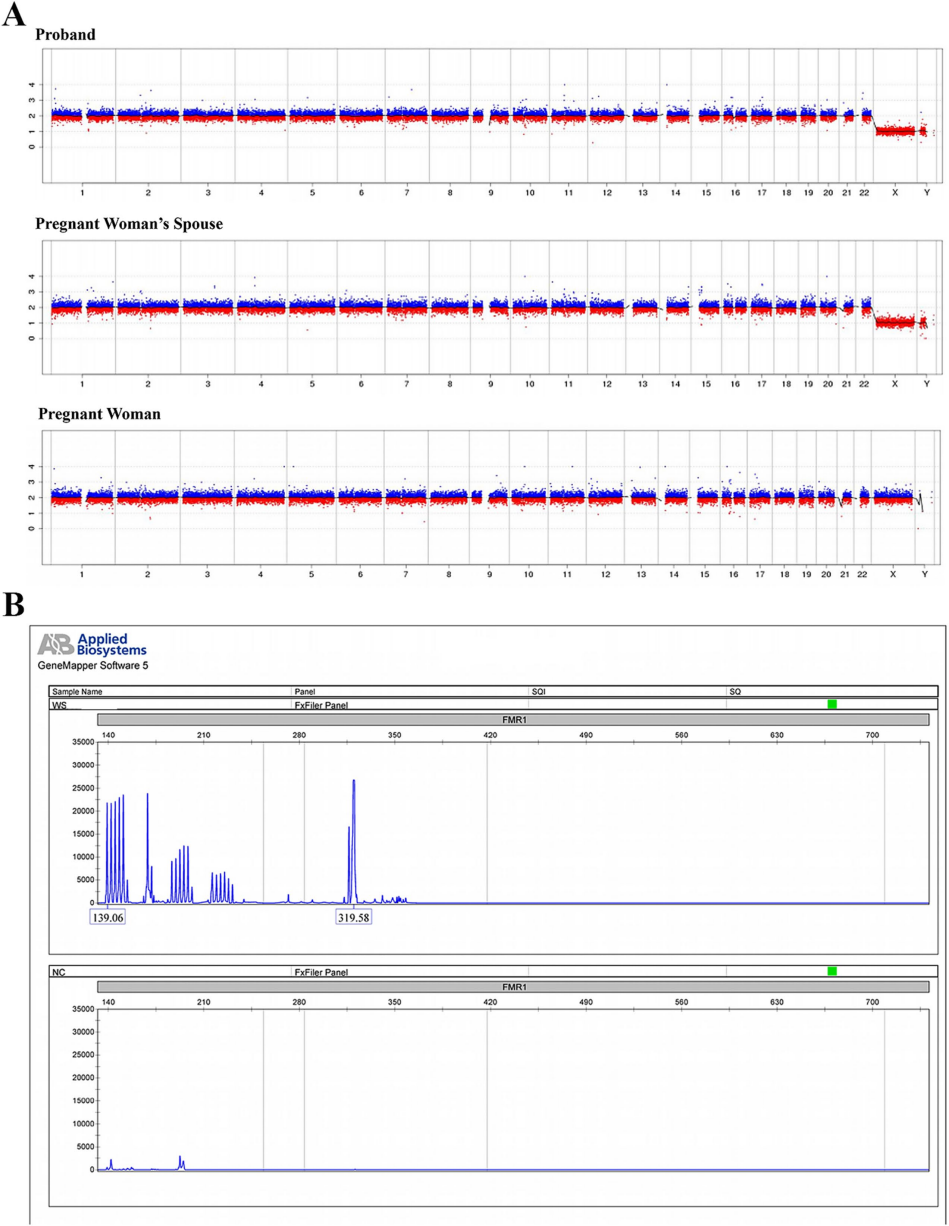
CNV sequencing and fragile X syndrome testing within the pedigree. **(A)** Low-coverage whole-genome sequencing based on high-throughput sequencing technology was performed to detect chromosomal aneuploidies or copy number variations (CNVs) ≥ 100 Kb in the proband, the pregnant woman’s spouse (father of the proband), and the pregnant woman (mother of the proband). **(B)** Fluorescent PCR combined with capillary electrophoresis was utilized to analyze the CGG repeat expansion in the FMR1 (fragile X mental retardation 1) gene of the proband.

Copy number variation sequencing (CNV-seq) was performed on NextSeq CN500 system with a depth sufficient for detecting copy number variations (CNVs) larger than 100 kb with a resolution of 0.1 Mb, capable of identifying aneuploidy and chromosomal mosaicism >10%. No clinically significant copy number variations were detected in the proband, the pregnant woman, or her spouse after referencing database of genomic variants (DGV) and ClinGen (Figure 3A). Fluorescent PCR coupled with capillary electrophoresis analysis of the FMR1 CGG repeat in the proband demonstrated 36 repeats, indicating low risk for fragile X syndrome (Figure 3B).

### 3.3 Prenatal counseling and follow-up

Following the genetic results, which indicated a male fetus for the current pregnancy with a high likelihood of being affected, the family were informed that the postnatal phenotype of the fetus would likely be similar to that of the older brother (the proband). Based on this information, the family ultimately decided to terminate the current pregnancy.

## 4 Discussion

This report presents the case of a pregnant woman with a history of adverse pregnancy outcomes. Through genetic analysis of the family pedigree, the genetic etiology of the proband was identified. In the subsequent pregnancy, amniocentesis was performed for prenatal genetic diagnosis, which determined the fetal genotype and enabled a prospective assessment of the postnatal disease risk. This critical information, combined with genetic counseling, informed the pregnancy management decision. Furthermore, this case revealed a previously unreported mutation in the NEXMIF gene, thereby expanding the mutational spectrum of this gene. To provide a comprehensive understanding of this case, we also present a detailed literature review on the associated disorder:

### 4.1 Genetic landscape of NEXMIF-related disorders

#### 4.1.1 Spectrum of NEXMIF variant

**Figure 4:**
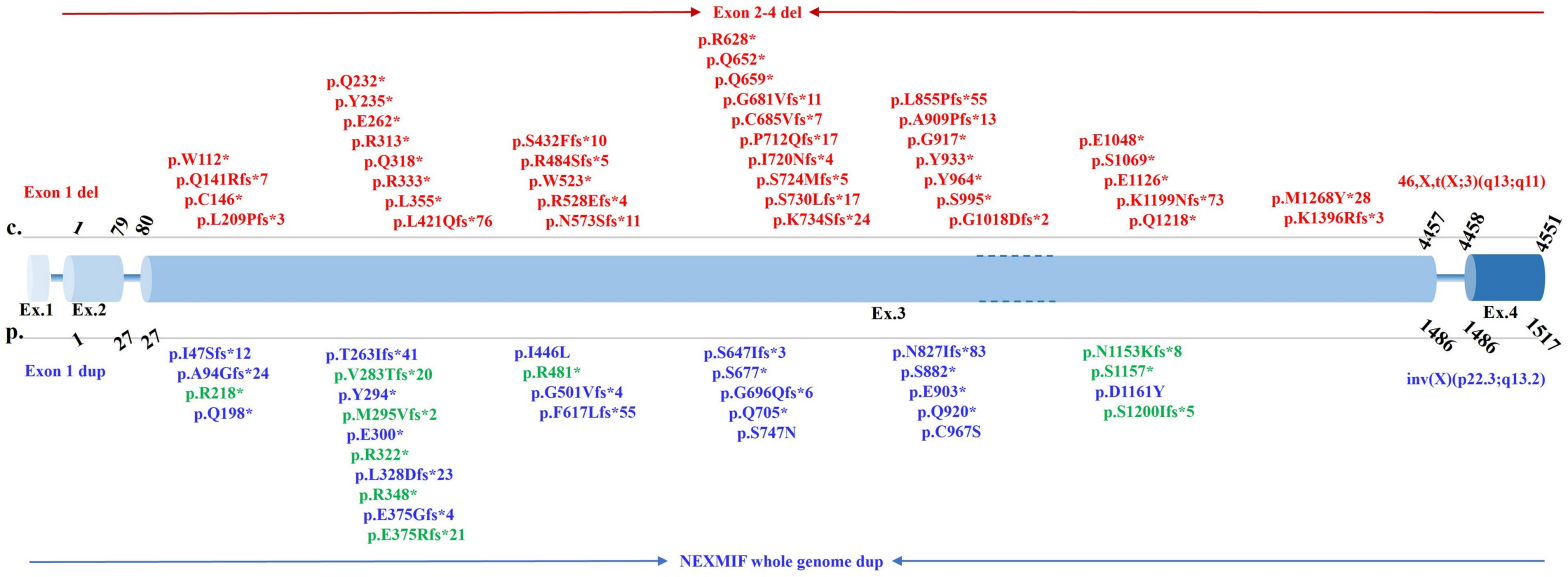
Spectrum of pathogenic NEXMIF variants within the genomic structure. This figure depicts pathogenic variants in NEXMIF identified in this case and published literature. Exon sizes are not drawn to scale. Variants reported exclusively in females are shown in red, those exclusively in males in blue, and variants reported in both sexes in green.

This review included a total of 77 types of variants, including pathogenic SNVs, InDels, gene deletions/duplications, as reported in the published literature (Supplementary Table S1). The vast majority of reported NEXMIF variants are loss-of-function mutations (nonsense and frameshift), with a remarkable and pronounced clustering within exon 3 (Figure 4). Large genomic deletions (6, 10, 11), duplications (4, 12), and rearrangements (5, 13, 14) are also significant contributors. Non-truncating (missense) variants are rare (reported cases (15) include p.I446L, p.S747N, p.C967S, p.D1161Y). Mosaicism has been documented for truncating variants (p.S882* at ~30% mosaicism (6); p.M295Vfs*2 at ~41% mosaicism (6); p.S677* at ~22.5% mosaicism (16)). The marked clustering of variants in exon 3 suggests that this region may harbor critical functional domains or exhibit heightened susceptibility to mutagenesis.

#### 4.1.2 Inheritance pattern and role of XCI

Research indicates that the majority of affected male patients inherit the pathogenic variant maternally, while female patients predominantly harbor *de novo* mutations (6, 15, 17, 18). The pathogenic mechanism of the NEXMIF gene is currently established as X-linked dominant inheritance (19). Key supporting evidence stems from the observation that most affected females exhibit random X-chromosome inactivation (XCI) (10) (Supplementary Table S1). Within families, sisters carrying the same mutation demonstrate varying patterns of random XCI or skewed XCI (6). Notably, the clinical symptoms appear largely comparable between females with random XCI and those with skewed XCI (6). However, reports exist of females with completely skewed (100%) XCI exhibiting a phenotype more akin to that of affected males (10, 20). Furthermore, studies suggest that individuals with skewed XCI are diagnosed with MAE-EMA syndrome without photosensitivity, whereas their sisters with random XCI exhibit a milder clinical phenotype (21). This indicates that XCI can explain, at least in part, the phenotypic differences between males and females, as well as the variability among female patients. Females with skewed XCI tend to present with a more severe phenotype, which can approach the severity seen in males. However, the relationship between XCI and clinical severity is complex. While skewed XCI can contribute to more severe phenotypes in females, its role is not absolute. The observed discrepancies between the XCI ratio in blood and symptom severity may be attributable to several factors. These include the inability of peripheral blood assays to accurately reflect the XCI status in brain tissue, and the potential for tissue-specific differences in NEXMIF expression levels, which could independently influence phenotypic expression.

#### 4.1.3 Genotype–phenotype correlations

Studies generally indicate a weak correlation between genotype and phenotype in NEXMIF-related disorders (6). Nevertheless, specific findings suggest potential associations. Firstly, mosaic male patients, whose mutational profile resembles that of female patients, tend to exhibit moderate intellectual disability and more severe seizure disorders compared to non-mosaic males (6). Secondly, systematic analysis reveals that patients harboring missense variants display a significantly higher frequency of normal intellectual development and a later age of seizure onset compared to patients carrying loss-of-function (nonsense or frameshift) variants (15). However, due to the relative scarcity of currently identified specific variant types, such as missense variants, further comprehensive analyses incorporating a broader spectrum of mutations are required to elucidate the genotype–phenotype relationship more definitively.

### 4.2 Clinical manifestations

Patients with pathogenic NEXMIF variants typically show no significant fetal abnormalities prenatally, though rare reports describe oligohydramnios (17), reduced/increased fetal movements (22, 23), or intrauterine growth restriction (IUGR) (24). In infancy, common features include hypotonia and poor visual tracking (19, 25, 26), with some cases developing infantile spasms (27, 28). Developmental milestones are stagnant or regressive with age (6, 17, 29), including intellectual, language, motor, and physical growth domains. Some individuals manifest severe allergies (24). Routine biochemical tests are generally normal (10), though thyroid dysfunction (25), gonadal dysregulation (6) and glucose abnormalities (30, 31) have been noted in some patients. Neuroimaging is often unremarkable (6, 10), but findings such as frontal lobe atrophy, temporopolar morphological changes, frontal cortical atrophy, and moderate brain atrophy (e.g., enlarged ventricles, prominent Virchow-Robin spaces, thin corpus callosum, small cerebellar vermis, and thick calvarium) have been reported (5, 6, 32). However, advanced neuroimaging techniques can detect thinning of the prefrontal cortex, particularly in the middle frontal gyrus, temporal cortex (including the fusiform gyrus), and pericalcarine visual cortex (33). This disorder is considered X-linked dominant with significant sex-based phenotypic differences. Males typically exhibit profound intellectual disability with or without seizure comorbidity, while females more frequently present with epilepsy and mild-to-moderate intellectual impairment. A detailed description of the multi-system clinical manifestations is provided below, with a comprehensive summary presented in Supplementary Figure S2.

#### 4.2.1 Nervous system

NEXMIF protein expression begins during fetal development and declines after birth with increasing age (34). The NEXMIF protein is predominantly expressed in brain tissue, with varying expression levels across different brain regions (Supplementary Figure S3), making neurological disorders the primary clinical manifestations associated with its dysfunction. Male patients typically present with moderate-to-severe intellectual disability (74%) (6), with or without epilepsy (35). Isolated epilepsy without intellectual impairment has also been reported (15). Female patients predominantly exhibit epilepsy (35), often accompanied by mild to moderate intellectual disability (67%) (6, 36). Seizure types commonly include poorly controlled generalized epilepsy with myoclonic and/or absence seizures (37, 38), myoclonic epilepsy (19), or Jeavons syndrome (21, 39, 40) (characterized by absence seizures with eyelid myoclonus, photosensitivity, and eye closure-induced paroxysmal EEG activity), but sudden unexpected death in epilepsy is rarely observed (41). All genders frequently demonstrate language dysfunction, limited to unintentional simple phrases (6, 35), alongside autism-like features such as stereotyped hand movements, poor social interaction (17, 25), hyperactivity (25, 42), behavioral abnormalities (self-injury, aggression) (4, 43), and mood disorders (anxiety, irritability) (17, 24). Notably, some female patients exhibit extroverted personalities and strong social skills (30). In one case, valproate administration triggered multiorgan dysfunction syndrome in a female patient, suggesting NEXMIF-related encephalopathy may predispose individuals to severe complications (9). Other neurological features include hypoalgesia (23), thermoregulatory dysfunction (23), sleep disturbances (insomnia, frequent awakening) (6), ataxia (6), and facial nerve palsy (37).

#### 4.2.2 Musculoskeletal system

The musculoskeletal presentation of NEXMIF-related disorders exhibits a spectrum of abnormalities, reflecting phenotypic heterogeneity among patients. Commonly reported findings include hypotonia (25), lower limb spasticity and muscular atrophy (4). In contrast, some cases have been documented with hypertonia and hyperreflexia (44), indicating variability in the motor phenotype. Additionally, transient jaw clenching during feeding and episodic hypertonia have been reported (23). Skeletal anomalies may include mild scoliosis/kyphosis, hypermobility of metacarpophalangeal/interphalangeal/large joints, pes planus, brachydactyly, and camptodactyly (12, 23). Neuromotor abnormalities include ataxic or wide-based gait, toe-walking, truncal instability, and heel cord tightness (4).

#### 4.2.3 Visual system

Ocular manifestations frequently involve strabismus (4, 17), astigmatism (44), severe bilateral keratoconus (23), and cortical visual impairment (23). While an isolated case of torpedo maculopathy has been reported in the context of an NEXMIF-related disorder (44), its significance is uncertain. Torpedo maculopathy is itself a rare condition, and this singular association has not been observed in larger patient cohorts (45). Consequently, it is currently unclear whether this represents a rare true association or a coincidental finding, underscoring the need for more evidence to clarify any potential link.

#### 4.2.4 Genitourinary/gastrointestinal systems

Gastroesophageal reflux disease is commonly observed and often contributes to refractory vomiting, feeding difficulties, and malnutrition in affected children (4, 25, 44). Other frequent gastrointestinal manifestations include dysmotility, constipation, dysphagia, and bloating (23). Beyond the gastrointestinal tract, urinary and fecal incontinence (23), recurrent urinary tract infections (17), and steroid-dependent nephrotic syndrome have been documented (25). Notably, excessive drooling has been described primarily in male patients (4, 5).

#### 4.2.5 Cardiac system

Most patients show no structural cardiac abnormalities on echocardiography (32). Rare reports include aortic stenosis, valvular dysplasia (46), atrial septal defect (16), mild mitral regurgitation (23), and cardiac rhabdomyoma (17).

#### 4.2.6 Reproductive system

A female patient with severe intellectual disability and primary amenorrhea was identified with a balanced X-autosome translocation (46, X, t (X; 3) (q13; q11)). At 24 years of age, persistent primary amenorrhea was noted; pelvic MRI revealed a hypoplastic uterus and non-visualized ovaries. This rearrangement disrupts the NEXMIF gene, suggesting a potential link between its dysfunction and impaired ovarian development/function (13).

#### 4.2.7 Physical examination

Growth parameters (height/weight) often fall below age-appropriate percentiles (22, 25), while some individuals develop adult-onset obesity (6). Characteristic craniofacial dysmorphism includes microcephaly (23, 46, 47), low-set ears (17), thick coarse hair (25), narrow forehead (22, 25), sunken nose (4, 37), short philtrum (24, 26), prognathism (23, 37), low hairline (17), wide-spaced teeth (6). Other features encompass hypertelorism, deep-set eyes, anteverted nostrils, upslanted palpebral fissures, bulbous nose, thin upper lip, thick vermilion lips and macroglossia (4, 6, 23, 26, 44). Cutaneous findings may involve café-au-lait spots (22, 44). A single case with cardiac rhabdomyoma and hypopigmented macules lacked TSC1/TSC2 mutations, suggesting a potential shared pathogenic mechanism between NEXMIF-related disorders and TSC1/TSC2-associated pathologies (17). Shawl scrotum was reported in one case (5).

### 4.3 Pathogenic mechanism

Initial studies demonstrate that siRNA-mediated NEXMIF knockdown significantly impairs neurite outgrowth in dendrites and axons, establishing its fundamental role in neuronal development (4). Mechanistically, subsequent research reveals that NEXMIF depletion leads to the transcriptional/translational upregulation of N-cadherin and β1-integrin. This, in turn, augments N-cadherin-mediated cell–cell adhesion and β1-integrin-dependent cell-matrix adhesion, which consequently impairs cellular migration in wound-healing assays (48). Notably, NEXMIF exhibits exclusive nuclear localization with developmentally restricted expression in cortical/subplate regions. Its knockdown disrupts neuronal migration and dendritic growth via N-cadherin/δ-catenin binding, which depletes cytosolic δ-catenin, elevates RhoA activity, and impairs actin dynamics—defects rescued by δ-catenin overexpression or RhoA inhibition (49). Complementary evidence from model organisms further elucidates this gene’s functions: In zebrafish, loss of NEXMIF truncates motor neuron axons and reduces branching, which is associated with downregulation of axon-guidance genes such as efna5b and sema6ba; these defects can be partially rescued by overexpression of the affected genes (50). Concurrently, murine models establish clinical relevance: NEXMIF KO mice recapitulate ASD phenotypes (social deficits, repetitive behaviors) and exhibit core synaptopathy, reduced dendritic spine density, decreased synaptic proteins (AMPAR/PSD-95/gephyrin), and suppressed transmission, confirming synaptic dysfunction drives behavioral deficits (51). At the circuit level, these mice show abolished behavior-dependent desynchronization in hippocampal CA1 networks, causing pathological hypersynchronization that disrupts neuronal coding precision (52). Critically advancing mechanistic understanding, mosaic models (NEXMIF ± mice) demonstrate that heterozygous insufficiency causes defective dendritic arborization and spine development in both NEXMIF-deficient and -expressing neurons, revealing dual cell-autonomous and non-cell-autonomous disruption of neural circuitry in ASD pathogenesis (53).

### 4.4 Synthesis and implications of the present case

In the context of the established literature, the present case both corroborates and refines the understanding of NEXMIF-related disorders. Our proband carries a novel hemizygous frameshift variant, which aligns with the predominance of loss-of-function mutations as the primary genetic lesion. The observed severe intellectual disability, profound language deficit, and motor delay in this male child are consistent with the typical neurodevelopmental phenotype described for affected males. However, the complete absence of epileptic seizures in our proband represents a notable deviation from the high prevalence of epilepsy reported in both male and female patients. This finding underscores the considerable phenotypic heterogeneity even among individuals with truncating variants and suggests that seizure susceptibility may be influenced by additional genetic or epigenetic modifiers not yet identified.

Furthermore, this family illustrates the variable expressivity characteristic of X-linked dominant disorders influenced by XCI. The mother, a heterozygous carrier of the same pathogenic variant, exhibits only mild intellectual disability with normal language and motor function. This mild presentation contrasts with the more severe phenotypes often observed in females with skewed X-inactivation and supports the model wherein random XCI in most carrier females can result in an attenuated, though still present, clinical manifestation. The lack of affected individuals in the wider family pedigree reinforces the *de novo* origin of this variant in the maternal germline, which is a common inheritance pattern for female carriers.

From a clinical translation perspective, this case powerfully demonstrates the critical role of precise genetic diagnosis in reproductive planning. The identification of the causative variant in this family enabled targeted prenatal diagnosis in the subsequent pregnancy, providing the parents with concrete information for risk assessment and informed decision-making. It also highlights the importance of considering NEXMIF in the differential diagnosis for males with severe, non-syndromic intellectual disability, even in the absence of epilepsy. Ultimately, this report expands the mutational spectrum of NEXMIF and contributes to a more nuanced view of its associated phenotypic range, emphasizing the need for individualized assessment and management in affected families.

## 5 Conclusion

In conclusion, this study underscores the critical importance of prenatal diagnosis via amniocentesis for assessing fetal disease risk in pregnancies following adverse obstetric histories. Furthermore, it provides a systematic review of NEXMIF-related disorders, yielding key insights: Firstly, the mutational spectrum is predominantly composed of truncating variants. While a clear genotype–phenotype correlation remains elusive, disease severity in females is often modulated by skewed X-chromosome inactivation (XCI), a relationship requiring further validation, as peripheral blood XCI may not reflect the brain. Secondly, clinical presentation is stratified by sex and developmental stage: prenatal findings are typically unremarkable; postnatally, intellectual disability is primary in males, whereas epilepsy predominates in females. Consequently, genetic testing for NEXMIF is crucial in patients presenting with intellectual disability and/or epilepsy to facilitate timely diagnosis and informed reproductive planning. Finally, although the precise pathogenic mechanisms are not fully elucidated, NEXMIF is known to be essential for synaptogenesis and cell adhesion pathway regulation. Future research should prioritize expanding patient cohorts, functionally characterizing diverse variants, and developing advanced models to decipher the underlying neurobiology.

## Data Availability

The original contributions presented in the study are included in the article/Supplementary material, further inquiries can be directed to the corresponding author.
