# Supplementary material for: Intellectual disorder type 98 caused by a novel NEXMIF variant: a case report and literature review
Source: Front Med (Lausanne). 2026 Feb 6;13:1757682. doi: 10.3389/fmed.2026.1757682 (PMC12920596; doi:10.3389/fmed.2026.1757682)
Supplement: Supplementary file 1 [file Data_Sheet_1.docx]

Table S1 Types of NEXMIF gene mutations in different families reported in the literature

| Affected Male | Affected Female | XCI |
| --- | --- | --- |
| c.1939_1942delinsAT, p.S647Ifs*3  NEXMIF dup^1^  inv(X)(p22.3;q13.2)^2^  c.3597dupA^3^  c.2113C>T, p.Q705X^4^  c.964C>T, p.R322X^4^  Exon 1 dup^3^  c.138 delC^3^  c.652C > T, p.Arg218* ^5^  c.2707G > T, p.Glu903* ^5^  c.3470C>A, p.Ser1157*^6^  c.1123delG, p.Glu375ArgfsX21^7^  c.1502delG, p.Gly501Valfs*4^8^  c.1848delC, p.Phe617fs^9^  p.Glu300*^10^  p.Ser882*, 30% mosaic^10^  p.Asn1153Lysfs*8^10^  p.Tyr294*^10^  p.Ala94Glyfs*24^10^  p.Gln920*^10^  p.Gly696Glnfs*6^10^  p.Met295Valfs*2, 41 % mosaic^10^  ChrX: 73565151–73956350^11^  c.788delC, p.T2631fsX41^12^  c.846_849delTGTC, p.V283TfsX20^12^  c.592C>T, p.Q198X^13^  c.652C>T, p.Arg218*^14^  c.2478_2479dup^15^  c.2030C>A, p.Ser677Ter, 22.5% mosaic^16^  c.1336A>C, p.Ile446Leu^17^  c.2240C>T, p.Ser747Asn^17^  c.2240C>T, p.Ser747Asn^17^  c.2889A>T, p.Cys967Ser^17^  c.3481C>A, p.Asp1161Tyr^17^  c.1441C>T, p.Arg481Ter^18^  c.982_c.983delTT, p.L328Dfs*23^19^  c.1123dupG, p. E375Gfs*4^20^  c.1042C > T, p. Arg348*^21^ | c.964C>T, p.R322X^22^  46,X,t(X;3)(q13;q11)^23^  c.4185del, p.Lys 1396fs^24^  c.438 C>A, p.Cys 146*^24^  c.2042del, p.Gly681fs^24^  c.964C>T, p.Arg322*^24^  c.2201_2202delAA, p.Lys734Serfs*24^24^  c.1441 C>T, p.Arg481*^24^  c.3053-3066del14, p.Gly1018Aspfs*2^24^  del exon1^24^  c.1582delA, p.Arg 528Glufs*4^24^  c.1882 C>T, p.Arg 628*^24^  c.2725del, p.Ala909Profs*13^24^  c.652C>T, p.Arg218*^24^  c.952C>T, p.Gln318*^24^  c.3596_3597 insA, p.Lys 1199Asnfs^24^  c.1882C>T, p.Arg628*^25^  c.3053_3066del14, p.G1018Dfsx2^26^  c.652 C > T, p.R218X^26^  c.422delA, p.Q141RfsX7^26^  c.625dupC, p.L209PfsX3^26^  c.C937T, p.R313X^26^  c.1718_1721del^27^  c.1063delC, p.L355*^28^  p.Arg481*^10^  p.Gln659*^10^  p.Tyr933*^10^  p.Arg322*^10^  p.Met1268Tyr*28^10^  p.Ile720Asnfs*4^10^  p.Arg628*^10^  p.Arg628*^10^  p.Glu1126*^10^  p.Leu421Glnfs*76^10^  p.Tyr964*^10^  p.Glu1048*^10^  p.Ser995*^10^  p.Pro712Glnfs*17^10^  p.Gly917*^10^  p.Trp112*^10^  p.Gln1218*^10^  p.Glu262*^10^  p.Gln652*^10^  p.Asn1153Lysfs* 8^10^  p.Asn1153Lysfs*8^10^  p.Arg628*^10^  p.Ser1200Ilefs*5^10^  p.Trp523*^10^  p.Arg218*^10^  ChrX:(73930523-74007913)x1^10^  p.Leu855Profs*55^10^  p.Arg481*^10^  p.Asn1153Lysfs*8^10^  p.Ser995*^10^  p.Trp112*^10^  p.Tyr964*^10^  p.Ser432Phefs*10^10^  p.Lys1199Asnfs*73^10^  p.Tyr235*^10^  p.Cys685Valfs*7^10^  p.Gln232*^10^  p.Met295Valfs*2^10^  c.1123del, p.Glu375Argfs*21^29^  c.2171delG, p.S724MfsTer5^30^  c.937C>T, p.Arg333*^12^  c.2189delC, p.S730Lfs*17^13^  c.3470C>A, p.Ser1157*^14^  c.937C>T, p.R313*^31^  c.1042C>T, p.Arg348Ter^17^  c.1449_1453delGAGAA, p.Arg484Serfs*5^17^  c.2171delG, p.Ser724fs^32^  c.846_849del, p.Val283Thrfs*20^33^  c.1882C>T, p.Arg628*^34^  c.3206C>A, p.Ser1069Ter^35^ | Random  Random  Random  Random  Random  Skewed  Random  Random  Skewed  Random  Random  Random/Skewed  Random  Random  Random/Skewed  Random  Random  Random  Random  Skewed  Random  Random  Random  Skewed  Random  Random  Random  Random  Random  Random |

**References**

**1.** Charzewska A, Rzońca S, Janeczko M, et al. A duplication of the whole KIAA2022 gene validates the gene role in the pathogenesis of intellectual disability and autism. Clin Genet 2015;88:297-9.

**2.** Cantagrel V, Lossi AM, Boulanger S, et al. Disruption of a new X linked gene highly expressed in brain in a family with two mentally retarded males. J Med Genet 2004;41:736-42.

**3.** Van Maldergem L, Hou Q, Kalscheuer VM, et al. Loss of function of KIAA2022 causes mild to severe intellectual disability with an autism spectrum disorder and impairs neurite outgrowth. Hum Mol Genet 2013;22:3306-14.

**4.** Kuroda Y, Ohashi I, Naruto T, et al. Delineation of the KIAA2022 mutation phenotype: two patients with X-linked intellectual disability and distinctive features. Am J Med Genet A 2015;167:1349-53.

**5.** Lorenzo M, Stolte-Dijkstra I, van Rheenen P, Smith RG, Scheers T, Walia JS. Clinical spectrum of KIAA2022 pathogenic variants in males: Case report of two boys with KIAA2022 pathogenic variants and review of the literature. Am J Med Genet A 2018;176:1455-62.

**6.** Lambert N, Dauve C, Ranza E, et al. Novel NEXMIF pathogenic variant in a boy with severe autistic features, intellectual disability, and epilepsy, and his mildly affected mother. J Hum Genet 2018;63:847-50.

**7.** Alarcon-Martinez T, Khan A, Myers KA. Torpedo Maculopathy Associated with NEXMIF Mutation. Mol Syndromol 2019;10:229-33.

**8.** Reuter MS, Chaturvedi RR, Liston E, et al. The Cardiac Genome Clinic: implementing genome sequencing in pediatric heart disease. Genet Med 2020;22:1015-24.

**9.** Panda PK, Sharawat IK, Joshi K, Dawman L, Bolia R. Clinical spectrum of KIAA2022/NEXMIF pathogenic variants in males and females: Report of three patients from Indian kindred with a review of published patients. Brain Dev 2020;42:646-54.

**10.** Stamberger H, Hammer TB, Gardella E, et al. NEXMIF encephalopathy: an X-linked disorder with male and female phenotypic patterns. Genet Med 2021;23:363-73.

**11.** Palmer EE, Sachdev R, Macintosh R, et al. Diagnostic Yield of Whole Genome Sequencing After Nondiagnostic Exome Sequencing or Gene Panel in Developmental and Epileptic Encephalopathies. Neurology 2021;96:e1770-1770e1782.

**12.** Langley E, Farach LS, Koenig MK, Northrup H, Rodriguez-Buritica DF, Mowrey K. NEXMIF pathogenic variants in individuals of Korean, Vietnamese, and Mexican descent. Am J Med Genet A 2022;188:1688-92.

**13.** Chen S, Deng X, Xiong J, et al. NEXMIF mutations in intellectual disability and epilepsy: A report of 2 cases and literature review. Zhong Nan Da Xue Xue Bao Yi Xue Ban 2022;47:265-70.

**14.** Stekelenburg C, Blouin JL, Santoni F, et al. Loss of Nexmif results in the expression of phenotypic variability and loss of genomic integrity. Sci Rep 2022;12:13815.

**15.** Chorny LE, Nordli DR 3rd, Galan F. NEXMIF Epilepsy: An Alternative Cause of Progressive Myoclonus. Neurology 2023;100:672-3.

**16.** Odgis JA, Gallagher KM, Rehman AU, et al. Detection of mosaic variants using genome sequencing in a large pediatric cohort. Am J Med Genet A 2023;191:699-710.

**17.** Ye ZL, Yan HJ, Guo QH, et al. NEXMIF variants are associated with epilepsy with or without intellectual disability. Seizure 2024;116:93-9.

**18.** Pande S, Majethia P, Nair K, et al. De novo variants underlying monogenic syndromes with intellectual disability in a neurodevelopmental cohort from India. Eur J Hum Genet 2024;32:1291-8.

**19.** He W, Liang Y, Yan H, Wan L, Yang G. [Clinical and genetic analysis of a child with West syndrome due to a de novo variant of NEXMIF gene]. Zhonghua Yi Xue Yi Chuan Xue Za Zhi 2024;41:725-9.

**20.** Li Z, Liu K, Zhao X, Li L. [Clinical and genetic analysis of a child with X-linked intellectual developmental disorder due to a novel variant of NEXMIF gene]. Zhonghua Yi Xue Yi Chuan Xue Za Zhi 2024;41:821-4.

**21.** Qi H, Pan D, Zhang Y, Zhu Y, Zhang X, Fu T. NEXMIF Combined with KIDINS220 Gene Mutation Caused Neurodevelopmental Disorder and Epilepsy: One Case Report. Actas Esp Psiquiatr 2024;52:588-94.

**22.** Farach LS, Northrup H. KIAA2022 nonsense mutation in a symptomatic female. Am J Med Genet A 2016;170:703-6.

**23.** Moysés-Oliveira M, Guilherme Rdos S, Dantas AG, et al. Genetic mechanisms leading to primary amenorrhea in balanced X-autosome translocations. Fertil Steril 2015;103:1289-96.e2.

**24.** de Lange IM, Helbig KL, Weckhuysen S, et al. De novo mutations of KIAA2022 in females cause intellectual disability and intractable epilepsy. J Med Genet 2016;53:850-8.

**25.** Athanasakis E, Licastro D, Faletra F, et al. Next generation sequencing in nonsyndromic intellectual disability: from a negative molecular karyotype to a possible causative mutation detection. Am J Med Genet A 2014;164A:170-6.

**26.** Webster R, Cho MT, Retterer K, et al. De novo loss of function mutations in KIAA2022 are associated with epilepsy and neurodevelopmental delay in females. Clin Genet 2017;91:756-63.

**27.** Samanta D, Willis E. KIAA2022-related disorders can cause Jeavons (eyelid myoclonia with absence) syndrome. Acta Neurol Belg 2020;120:205-7.

**28.** Wu D, Ji C, Chen Z, Wang K. Novel NEXMIF gene pathogenic variant in a female patient with refractory epilepsy and intellectual disability. Am J Med Genet A 2020;182:2765-72.

**29.** Ogasawara M, Nakagawa E, Takeshita E, et al. Clonazepam as an Effective Treatment for Epilepsy in a Female Patient with NEXMIF Mutation: Case Report. Mol Syndromol 2020;11:232-7.

**30.** Cioclu MC, Coppola A, Tondelli M, et al. Cortical and Subcortical Network Dysfunction in a Female Patient With NEXMIF Encephalopathy. Front Neurol 2021;12:722664.

**31.** Wang L, Huang Y, Liu X. NEXMIF pathogenic variant in a female child with epilepsy and multiple organ failure: a case report. Transl Pediatr 2023;12:1278-87.

**32.** Coppola A, Krithika S, Iacomino M, et al. Dissecting genetics of spectrum of epilepsies with eyelid myoclonia by exome sequencing. Epilepsia 2024;65:779-91.

**33.** Badura-Stronka M, Wołyńska K, Winczewska-Wiktor A, et al. Validation of targeted next-generation sequencing panels in a cohort of Polish patients with epilepsy: assessing variable performance across clinical endophenotypes and uncovering novel genetic variants. Front Neurol 2023;14:1316933.

**34.** Anastasescu CM, Gheorman V, Godeanu SV, et al. KIAA2022/NEXMIF c.1882C>T (p.Arg628*) Variant in a Romanian Patient with Neurodevelopmental Disorders and Epilepsy: A Case Report and Systematic Review. Life (Basel) 2025;15:497.

**35.** Adarsha N, Sait H. Gonadal mosaicism and paradoxical phenotype in NEXMIF encephalopathy: a case report of two siblings. J Genet 2025;104:6 [pii].


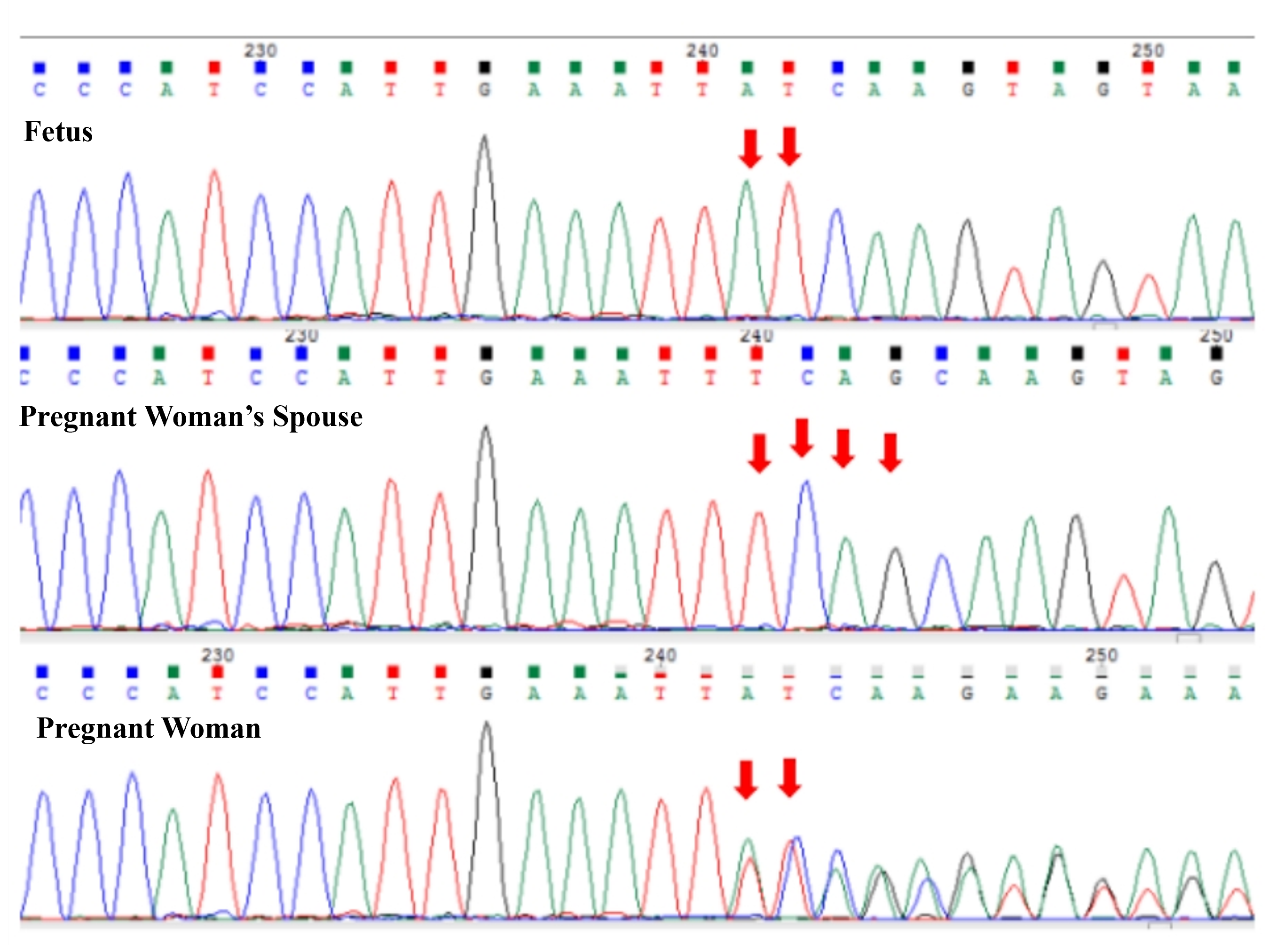


Figure S1 Sanger sequencing confirmation of the NEXMIF c.1939_1942delinsAT variant in the fetus.


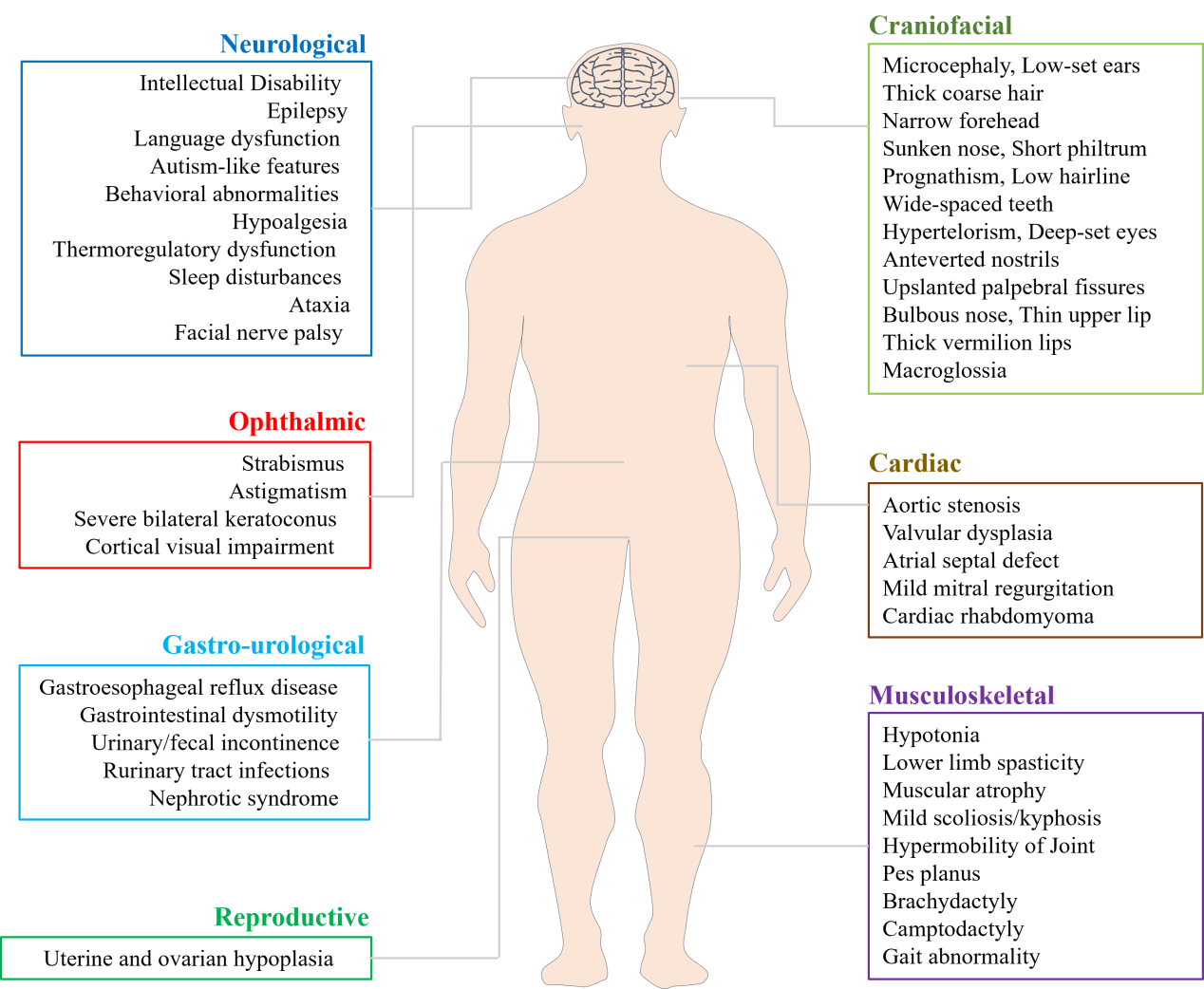


Figure S2 Clinical phenotypes of affected individuals with intellectual developmental disorder type 98.


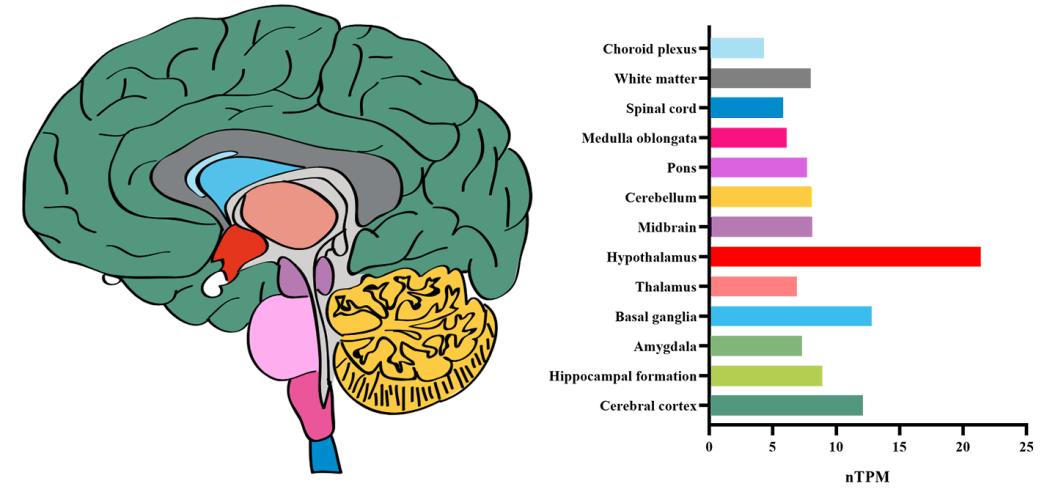


Figure S3 Regional Brain Expression Profile of Normalized NEXMIF RNA Levels. Data analyzed from 13 brain regions using the Human Protein Atlas (HPA) database. The resulting normalized transcript expression values, denoted nTPM, were calculated for each gene in every sample.
